# Supplementary material for: Ets transcription factor GABP controls T cell homeostasis and immunity
Source: Nat Commun. 2017 Oct 20;8:1062. doi: 10.1038/s41467-017-01020-6 (PMC5648787; doi:10.1038/s41467-017-01020-6)
Supplement: Supplementary file 1 — Supplementary Information [file 41467_2017_1020_MOESM1_ESM.pdf]

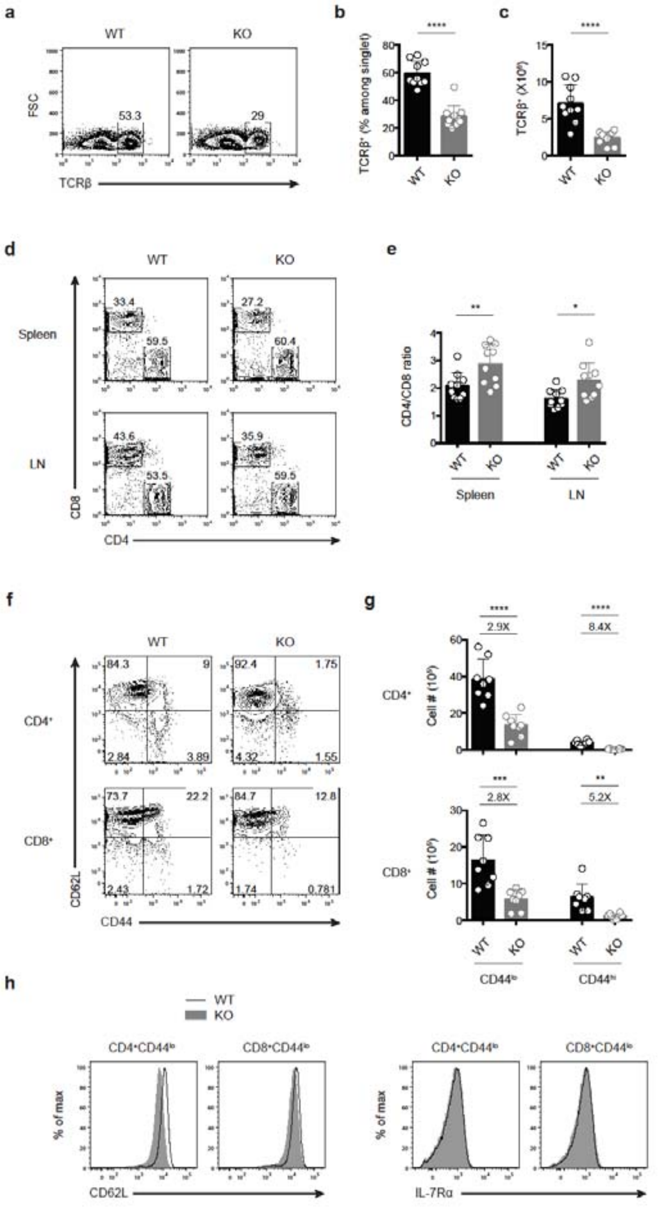

Supplementary Figure 1: Diminished peripheral T cell populations in the T cell-specific GABPα-deficient mice. **a**, Flow cytometric analysis of TCRβ expression on cells from peripheral lymph nodes (LNs) of *Gabpa*$^{f/f}$ (wild-type, WT) and *CD4*^{Cre}*Gabpa*$^{f/f}$ (KO) mice. **b-c**, Fractions of TCRβ^{+} cells among total live cells (**b**), and numbers of TCRβ^{+} cells (**c**) in the LNs of WT and KO mice. **d**, Expression of CD4 and CD8 on TCRβ^{+} cells from spleen and peripheral LNs of WT and KO mice. **e**, Ratio of CD4^{+} to CD8^{+} cells. **f**, Flow cytometric analysis of CD44 and CD62L expression on CD4^{+} and CD8^{+} T cells from peripheral LNs of WT and KO mice. **g**, Numbers of CD44^{lo} and CD44^{hi} subsets of CD4^{+} and CD8^{+} in the LNs of WT and KO mice. Fold changes comparing KO to WT are shown above the plots. **h**, Expression of CD62L and IL-7Rα in CD44^{lo} naive CD4^{+} and CD8^{+} T cells from LNs of WT and KO mice. Mice of 5-8 week-old were used. Data represent 8-10 mice per genotype analyzed in at least three independent experiments (mean ± SEM; unpaired *t*-test).

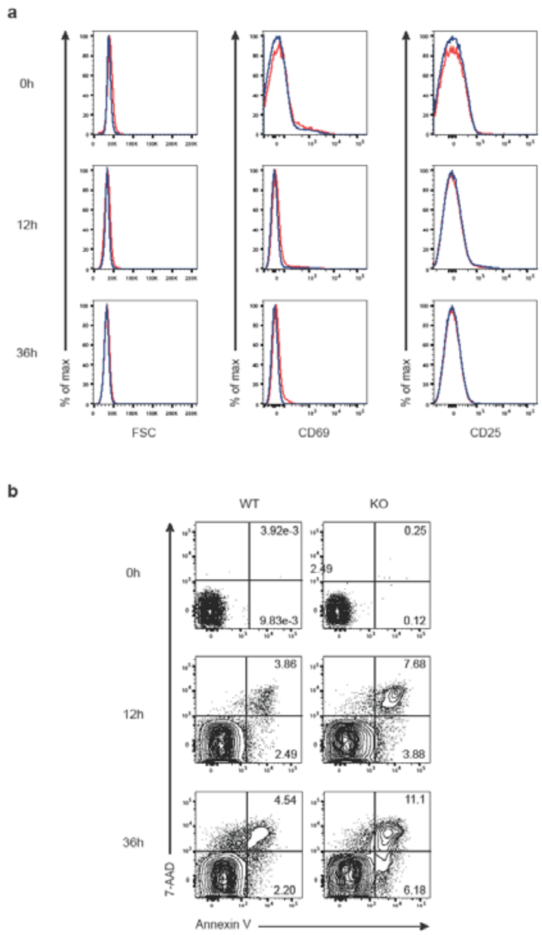

Supplementary Figure 2: GABPα is required for naïve T cell survival *in vitro*. Naive ($CD62L^{hi}$ $CD44^{lo}$) $CD4^+$ or $CD8^+$ T cells from *Gabpa*$^{f/f}$ (WT) and *CD4*^{Cre}*Gabpa*$^{f/f}$ (KO) mice were purified by flow cytometric sorting, and were cultured in the presence of IL-7. Representative plots from $CD8^+$ T cell culture were shown. **a**, Analysis of cell size (FSC) and activation markers, CD69 and CD25, at 0h, 12h and 36h post stimulation. **b**, Cell death was assessed with Annexin V and 7-AAD staining. Data represent at least three independent experiments.

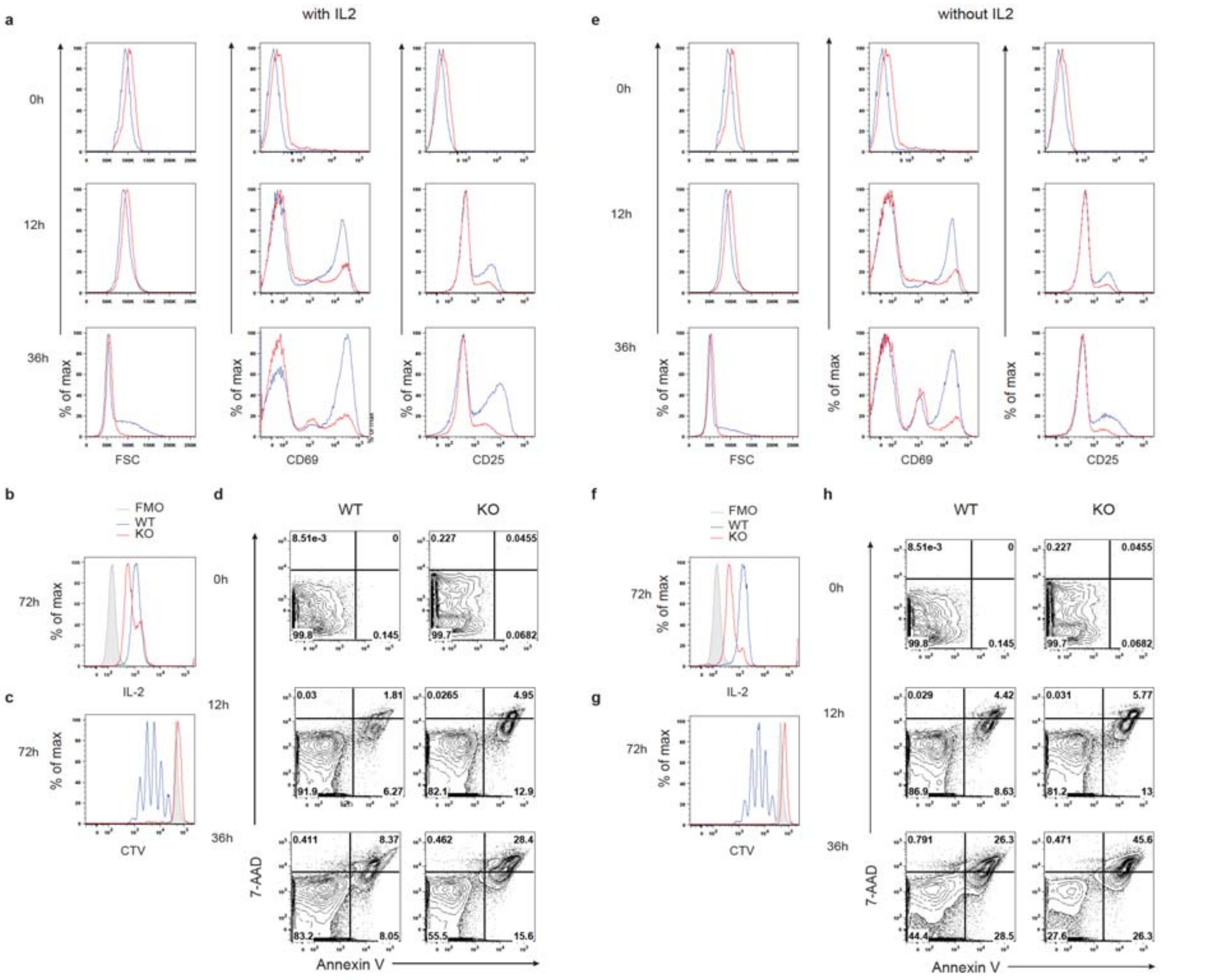

Supplementary Figure 3: GABPα is required for T cell activation, proliferation and survival in response to antigen stimulation *in vitro*. Naive (CD62L^{hi} CD44^{lo}) CD4^{+} or CD8^{+} T cells from *Gabpa$^{f/f}$* (WT) and *CD4^{Cre}Gabpa$^{f/f}$* (KO) mice were purified by flow cytometric sorting, and were subjected to anti-CD3 and CD28 stimulation in the presence **(a-d)** or absence **(e-h)** of IL-2. Representative plots from CD8^{+} T cell culture were shown. **a**, **e**, Analysis of cell size (FSC) and activation markers, CD69 and CD25, at 0h, 12h and 36h post stimulation. **b**,**f**, 72h after cell culture, WT and KO cells were restimulated with PMA and ionomycin for 4h and analyzed for the expression of IL-2 by intracellular cytokine staining. **c**,**g**, WT and KO cells were labeled with the cytosolic dye CFSE, and cell division was assessed by the dilution of CFSE. Grey shaded line shows CFSE level of undivided cells. **d**, **h**, Cell death was assessed with Annexin V and 7-AAD staining.

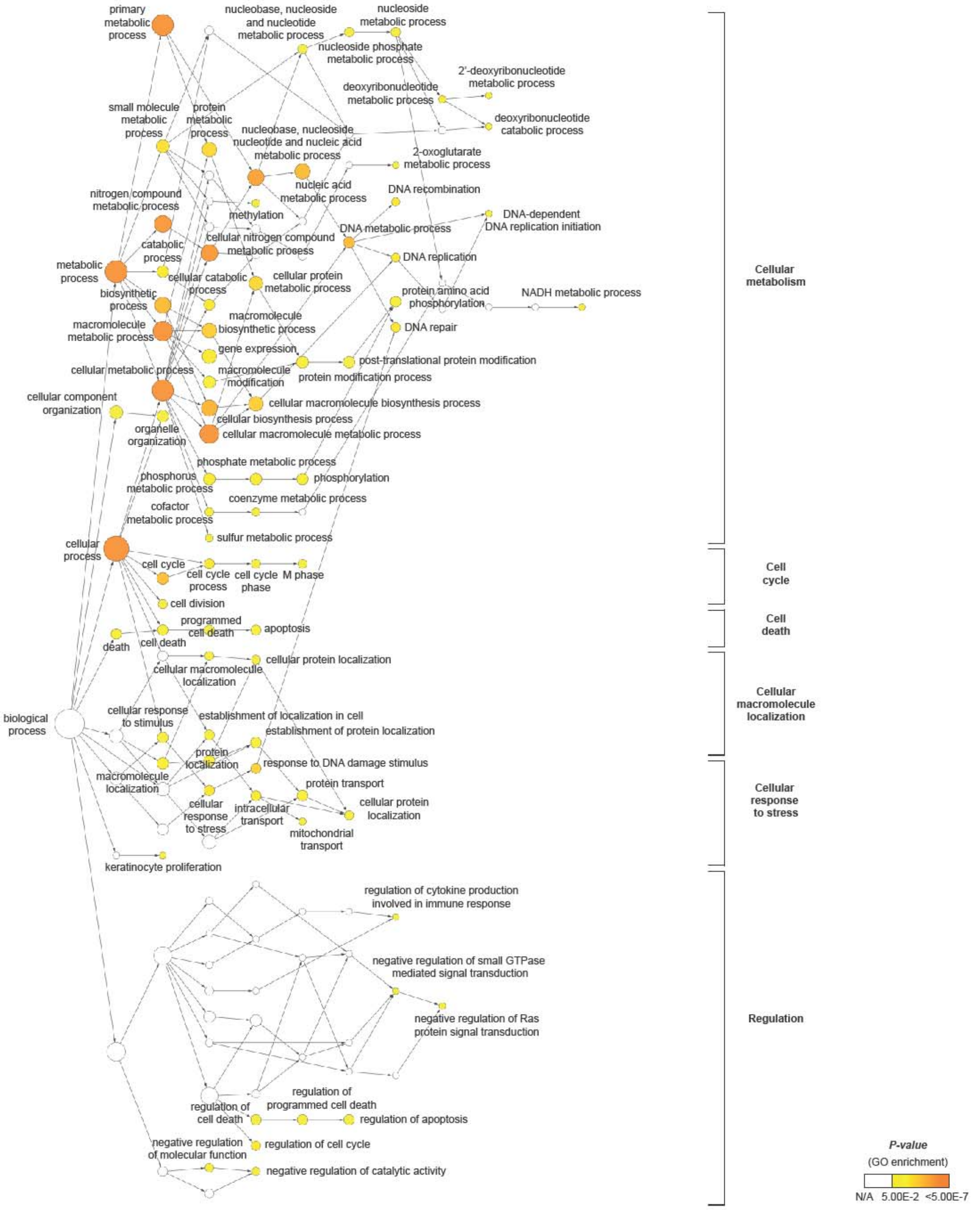

Supplementary Figure 4: Gene ontology (GO) analysis of GABPα-regulated genes. GABPα-regulated genes (Supplementary Table 1) were defined as: 1) differentially expressed between GABPα-sufficient and -deficient T cells at 0-hour or 18-hour time point or both; 2) expression changes were consistent between $CD4^+$ and $CD8^+$ T cells. The GO analysis was performed in Cytoscape using the BiNGO plug-in version 3.0.3. Presented is a reduced network showing all biological process categories that were significantly over-represented, with the color scale indicates the level of significance (adjusted p<0.05). The size of the circles is proportional to the number of genes in each category.

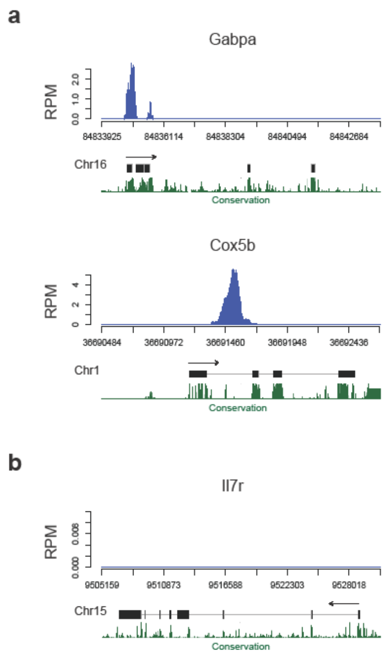

Supplementary Figure 5: Analysis of GABPα-bound target genes using ChIP-seq. a, GABPα-bound regions for several previously characterized GABP target genes, *Gabpa and Cox5b*. **b**, ChIP-seq result of *Il7r* gene locus. Gene structure, chromosomal location, and sequence homology were shown for each region. The Y-axis represent read per million (RPM).

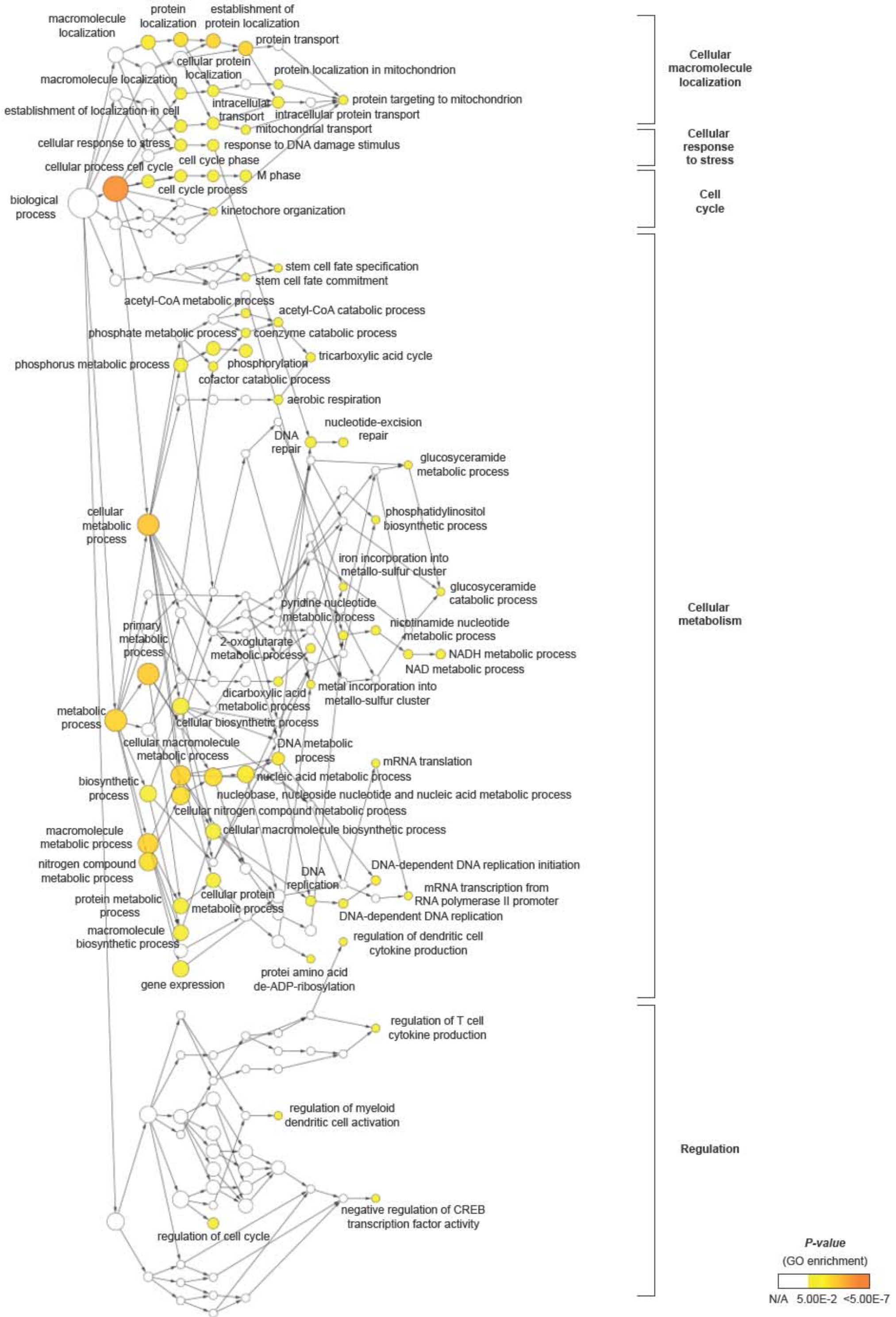

Supplementary Figure 6: Gene ontology (GO) analysis of GABPα direct target genes. GABPα direct target genes (Supplementary Table 4) were defined as: 1) differentially expressed between wild-type and GABPα knockout T cells at 0-hour or 18-hour time point or both; 2) expression changes were consistent between $CD4^+$ and $CD8^+$ T cells; 3) GABPα was recruited

to the gene locus in the ChIP-seq experiments. The GO analysis was performed in Cytoscape using the BiNGO plug-in version 3.0.3. Presented is a reduced network showing all biological process categories that were significantly over-represented, with the color scale indicates the level of significance (adjusted $p<0.05$). The size of the circles is proportional to the number of genes in each category.

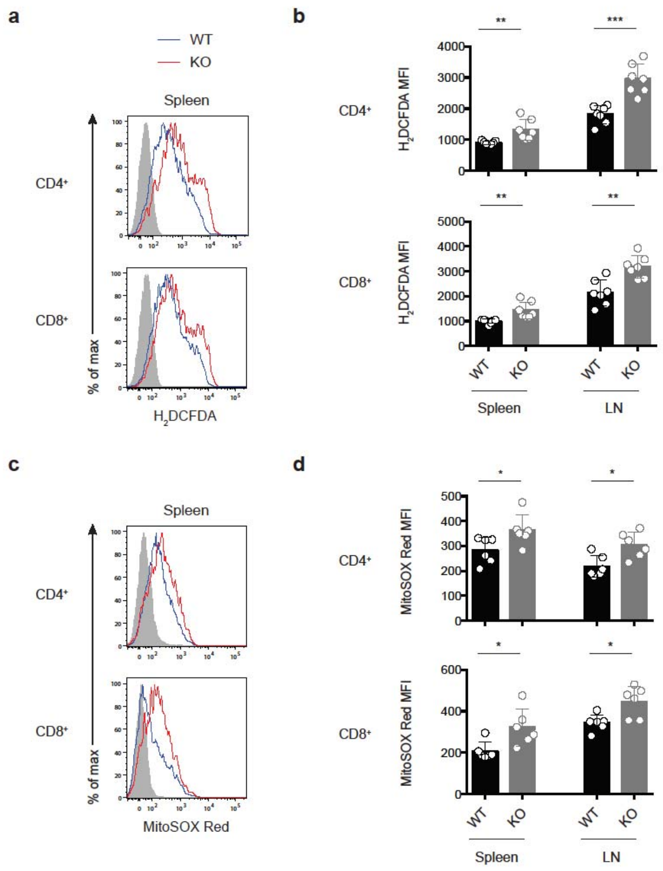

Supplementary Figure 7: GABPα maintains cellular redox homeostasis. $CD4^+$ or $CD8^+$ T cells from spleen and lymph nodes (LNs) of 5-8 week-old *Gabpa*$^{f/f}$ (WT) and *CD4*^{Cre}*Gabpa*$^{f/f}$ (KO) mice were analyzed. **a-b**, Representative flow cytometric plots of cellular ROS level assessed by H_2DCFDA staining **(a)** and the mean fluorescence index (MFI) **(b)** were shown. **c-d**, Ex vivo analysis of mitochondrial ROS level by MitoSOX Red staining **(c)** and the MFI of MitoSOX Red **(d)** were shown. Data represent 6-7 mice per genotype analyzed in at least three independent experiments (mean ± SEM; unpaired *t*-test).

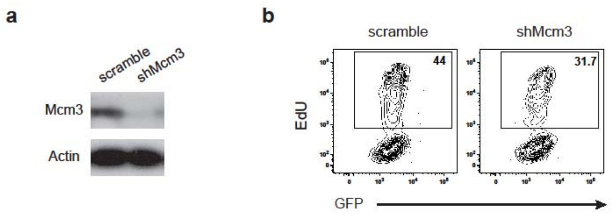

Supplementary Figure 8: Knockdown of Mcm3 reduces T cell proliferation. a Immunoblot analysis of Mcm3 knockdown by shMcm3 in wild-type $CD8^+$ T cells. Scramble shRNA was used as control. **b**, Flow cytometric analysis of EdU incorporation in the scramble and shMcm3 expressing $CD8^+$ T cells.

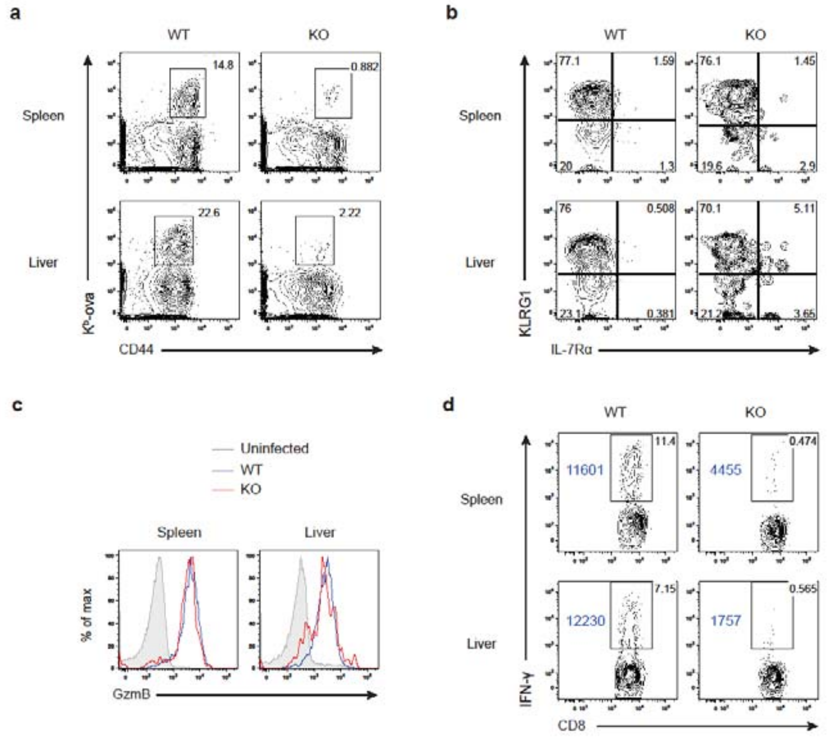

Supplementary Figure 9: Phenotypic analysis ovalbumin-specific cells in GABPα-deficient mice post *L. monocytogene*s infection. *Gabpa*$^{f/f}$ (WT) and *CD4*^{Cre}*Gabpa*$^{f/f}$ (KO) mice were infected with LM-OVA. At Day 7-post infection, OVA-specific CD8$^+$ T cell responses were analyzed by the staining of K^b-ova tetramer. **a**, Representative flow cytometric blots of CD4 and K^b-ova staining in CD8$^+$ T cells from spleen and liver. **b**, Flow cytometric analysis of KLRG-1 and IL-7Rα expression of K^b-ova$^+$ CD8$^+$ T cells from spleen and liver. **c**, Granzyme B (GzmB) expression in K^b-ova$^+$ CD8$^+$ T cells from spleen and liver. **d**, Production of IFN-γ in WT and KO CD8$^+$ T cells after restimulation with SIINFEKL peptide. Percentage of IFN-γ$^+$ cells of CD8$^+$ T cells was shown in black, and IFN-γ MFI was shown in blue.

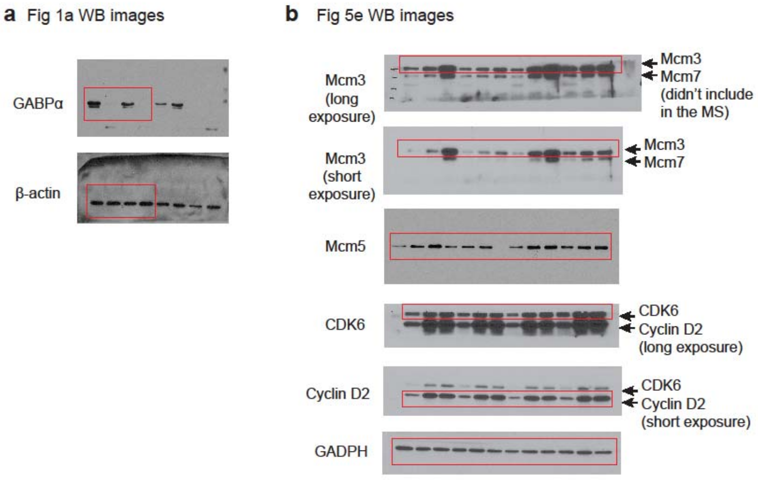

Supplementary Figure 10: Uncropped images for immunoblotting experiments shown in the figures.
