# Supplementary material for: Ets transcription factor GABP controls T cell homeostasis and immunity
Source: Nat Commun. 2017 Oct 20;8:1062. doi: 10.1038/s41467-017-01020-6 (PMC5648787; doi:10.1038/s41467-017-01020-6)
Supplement: Supplementary file 3 — Description of Additional Supplementary Files [file 41467_2017_1020_MOESM3_ESM.pdf]

Description of Additional Supplementary Files

File Name: Supplementary Data 1
Description: Differentially expressed genes in GABPa-deficient T cells (1.5X or greater, either 0hr or 18hr, shared between CD4 and CD8)

File Name: Supplementary Data 2
Description: Gene ontology analysis of GABPa-regulated genes (differentially expressed genes listed in Supplementary Table 1)

File Name: Supplementary Data 3
Description: Complete list of candidate binding sites detected in GABPa ChIP-Seq

File Name: Supplementary Data 4
Description: Complete list of putative direct target genes of GABPa

File Name: Supplementary Data 5
Description: Gene ontology analysis of GABPa direct target genes
